# Continuous terlipressin versus vasopressin infusion in septic shock (TERLIVAP): a randomized, controlled pilot study

**DOI:** 10.1186/cc7990

**Published:** 2009-08-10

**Authors:** Andrea Morelli, Christian Ertmer, Sebastian Rehberg, Matthias Lange, Alessandra Orecchioni, Valeria Cecchini, Alessandra Bachetoni, Mariadomenica D'Alessandro, Hugo Van Aken, Paolo Pietropaoli, Martin Westphal

**Affiliations:** 1 Department of Anesthesiology and Intensive Care, University of Rome, "La Sapienza", Viale del Policlinico 155, Rome 00161, Italy; 2 Laboratory of Clinical Pathology, Department of Surgery, University of Rome, "La Sapienza", Viale del Policlinico 155, Rome 00161, Italy; 3 Department of Anesthesiology and Intensive Care, University Hospital of Muenster, Albert-Schweitzer-Strasse 33, Muenster 48149, Germany

## Abstract

**Introduction:**

Recent clinical data suggest that early administration of vasopressin analogues may be advantageous compared to a last resort therapy. However, it is still unknown whether vasopressin and terlipressin are equally effective for hemodynamic support in septic shock. The aim of the present prospective, randomized, controlled pilot trial study was, therefore, to compare the impact of continuous infusions of either vasopressin or terlipressin, when given as first-line therapy in septic shock patients, on open-label norepinephrine requirements.

**Methods:**

We enrolled septic shock patients (n = 45) with a mean arterial pressure below 65 mmHg despite adequate volume resuscitation. Patients were randomized to receive continuous infusions of either terlipressin (1.3 μg·kg^-1^·h^-1^), vasopressin (.03 U·min^-1^) or norepinephrine (15 μg·min^-1^; n = 15 per group). In all groups, open-label norepinephrine was added to achieve a mean arterial pressure between 65 and 75 mmHg, if necessary. Data from right heart and thermo-dye dilution catheterization, gastric tonometry, as well as laboratory variables of organ function were obtained at baseline, 12, 24, 36 and 48 hours after randomization. Differences within and between groups were analyzed using a two-way ANOVA for repeated measurements with group and time as factors. Time-independent variables were compared with one-way ANOVA.

**Results:**

There were no differences among groups in terms of systemic and regional hemodynamics. Compared with infusion of .03 U of vasopressin or 15 μg·min^-1 ^of norepinephrine, 1.3 μg·kg^-1^·h^-1 ^of terlipressin allowed a marked reduction in catecholamine requirements (0.8 ± 1.3 and 1.2 ± 1.4 vs. 0.2 ± 0.4 μg·kg^-1^·min^-1 ^at 48 hours; each *P *< 0.05) and was associated with less rebound hypotension (*P *< 0.05). At the end of the 48-hour intervention period, bilirubin concentrations were higher in the vasopressin and norepinephrine groups as compared with the terlipressin group (2.3 ± 2.8 and 2.8 ± 2.5 vs. 0.9 ± 0.3 mg·dL^-1^; each *P *< 0.05). A time-dependent decrease in platelet count was only observed in the terlipressin group (*P *< 0.001 48 hours vs. BL).

**Conclusions:**

The present study provides evidence that continuous infusion of low-dose terlipressin – when given as first-line vasopressor agent in septic shock – is effective in reversing sepsis-induced arterial hypotension and in reducing norepinephrine requirements.

**Trial registration:**

ClinicalTrial.gov NCT00481572.

## Introduction

In the past few years, it has become evident that the efficacy of hemodynamic optimization by fluids and vasopressor agents critically depends on the urgency of therapy [1-4]. The recent Vasopressin and Septic Shock Trial (VASST) [5] revealed that survival was only improved in the subgroup of patients receiving vasopressin (AVP) in the less severe state of disease, as indicated by low doses of norepinephrine (NE) infusion (i.e. ≤15 μg/min) prior to randomization. In some European countries, however, AVP is not available, and thus terlipressin (TP), a synthetic, long-acting vasopressin analogue, is commonly considered as last resort therapy in the late phase of septic shock, when high dosages of catecholamines fail to counteract sepsis-related arterial hypotension [6-9]. Due to its long effective half-life of four to six hours, TP is commonly administered as high-dose bolus infusion (about 1 mg every four to six hours). The potential problem, however, is that TP bolus infusion may contribute to excessive vasoconstriction and a reflectory decrease in cardiac output with a proportional depression in oxygen delivery [10]. This may be especially problematic in a condition of increased oxygen demand, such as early sepsis [1,3]. Notably, preliminary experimental and clinical reports have shown that TP may also be administered as low-dose continuous infusion, thereby mitigating, or even preventing such adverse events [10-14]. The optimal time of therapy, however, remains to be determined.

Preliminary results from a comparative experimental study of AVP versus TP in ovine septic shock suggested that continuous infusion of TP may improve survival and increase mesenteric perfusion as compared with AVP [15]. In addition, it has been reported that a highly selective V_1 _agonist (FE 202158) markedly reduced vascular leakage and mortality in experimental sepsis as compared with AVP [16,17]. Nevertheless, a direct comparison between a continuous infusion of a relatively selective V_1 _agonist, such as TP, and AVP on catecholamine requirements in human septic shock has not yet been performed. We hypothesized that the relatively selective V_1 _receptor agonist TP is likewise advantageous when compared with AVP in human septic shock.

Therefore, we conducted a randomized controlled clinical pilot study to compare the effects of first-line institution of continuous, fixed doses of TP and AVP infusion on open-label NE requirements in patients with septic shock. In addition, we aimed to investigate the effects of both vasopressor agents on systemic and regional hemodynamics as well as organ function.

## Materials and methods

### Patients

After approval by the Local Institutional Ethics Committee, the study was performed in an 18-bed multidisciplinary intensive care unit (ICU) of the Department of Anesthesiology and Intensive Care of the University of Rome 'La Sapienza'. Due to the protocol design, informed consent was obtained from the patients' next of kin at the time of ICU admission. Enrolment of patients started in January 2007 and ended in January 2008. We enrolled patients who fulfilled the criteria of septic shock [3] presenting with a mean arterial pressure (MAP) below 65 mmHg despite appropriate volume resuscitation (pulmonary arterial occlusion pressure (PAOP) = 12 to 18 mmHg and central venous pressure = 8 to 12 mmHg) [3] during the ICU stay.

Exclusion criteria were age less than 18 years, catecholamine therapy prior to randomization, pronounced cardiac dysfunction (i.e. cardiac index ≤2.2 L/min/m in the presence of PAOP > 18 mmHg), chronic renal failure, severe liver dysfunction (Child-Turcotte-Pugh grade C), significant valvular heart disease, present coronary artery disease, pregnancy, and present or suspected acute mesenteric ischemia or vasospastic diathesis (e.g. Raynaud's syndrome or related diseases).

All patients were sedated with sufentanil and midazolam and received mechanical ventilation using a volume-controlled mode.

### Measurements

Systemic hemodynamic monitoring of the patients included a pulmonary artery catheter (7.5-F, Edwards Lifesciences, Irvine, CA, USA) and a radial artery catheter. MAP, right atrial pressure (RAP), mean pulmonary arterial pressure (MPAP), and PAOP were measured at end-expiration. Heart rate (HR) was analyzed from a continuous recording of electrocardiogram with ST segments monitored. Cardiac index (CI) was measured using the continuous thermodilution technique (Vigilance II^®^, Edwards Lifesciences, Irvine, CA, USA). Arterial and mixed-venous blood samples were taken to determine oxygen tensions and saturations, as well as carbon dioxide tensions, standard bicarbonate and base excess. Mixed-venous oxygen saturation (SvO_2_) was measured discontinuously by intermittent mixed-venous blood gas analyses. Systemic vascular resistance index (SVRI), pulmonary vascular resistance index (PVRI), left and right ventricular stroke work indices (LVSWI, RVSWI), systemic oxygen delivery index (DO_2_I), oxygen consumption index (VO_2_I), and oxygen extraction ratio (O_2_-ER) were calculated using standard formulae.

Regional hemodynamic monitoring was performed using a 4-F oximetry thermo-dye dilution catheter (PV2024L, Pulsion Medical System AG, Munich, Germany) inserted into the femoral artery for the measurement of plasma disappearance rate (PDR) and blood clearance of indocyanine green related to body surface area (CBI). PDR and CBI were determined with the Cold Z-021 system (Pulsion Medical System AG, Munich, Germany) using an established protocol [18,19]. In addition, an air-tonometer (Tonocap, Datex-Ohmeda, Helsinki, Finland) was inserted via the naso-gastric route for measurement of gastric mucosal carbon dioxide partial pressure and calculation of the gradient between gastric mucosal and partial pressure of arterial carbon dioxide [20,21].

Arterial blood samples were drawn and analyzed for pH, arterial lactate, aspartate aminotransferase, alanine aminotransferase, total bilirubin (BILT), direct bilirubin (BILD), amylase, lipase, international normalized ratio, activated partial thromboplastin time ratio, cardiac troponin I, TNF-α, IL-1β, and IL-6. Urine samples were collected to assess urinary output and creatinine clearance.

### Study design

**Figure 1:**
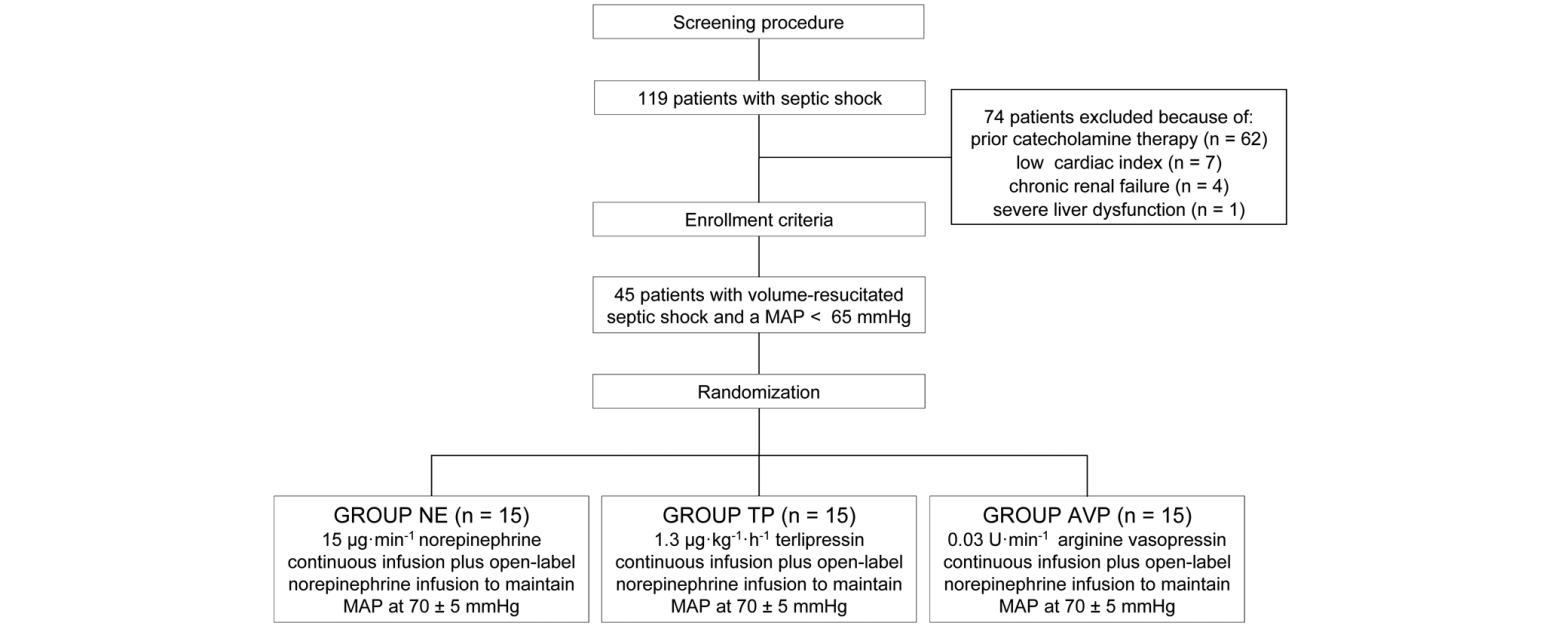
Study design. AVP = arginine vasopressin; MAP = mean arterial pressure; NE = norepinephrine; TP = terlipressin.

Patients were randomized to one of three study groups using a computer-based procedure. Patients allocated to the TP group received a continuous TP infusion of 1.3 μg/kg/hour and patients in the AVP group were treated with a continuous infusion of AVP at 0.03 U/min. The control group received a fixed dose of NE (15 μg/min). In all three groups, open-label NE was additionally infused, if the goal MAP of 70 ± 5 mmHg was not achieved with study drug infusion alone (Figure 1).

Fluid challenge was performed to maintain central venous pressure at 8 to 12 mmHg and PAOP between 12 and 18 mmHg during the 48-hour intervention period [3]. Packed red blood cells were transfused when hemogloblin concentrations decreased below 8 g/dL. If SvO_2 _was less than 65% despite appropriate arterial oxygenation (arterial oxygen saturation ≥95%) and hemoglobin concentrations wer 8 g/dL or above, dobutamine was administered in doses up to 20 μg/kg/min to achieve SvO_2 _values of 65% or more, if possible [3]. During the 48-hour study period, all patients received intravenous hydrocortisone (200 mg/day) as a continuous infusion.

Systemic, pulmonary, and regional hemodynamic measurements, laboratory variables, blood gases as well as NE requirements, were determined at baseline, 12, 24, 36 and 48 hours after randomization. Plasma cytokine concentrations were measured at baseline and after 48 hours.

In patients surviving the 48-hour intervention period, study drug infusion was terminated, and open-label NE was titrated to maintain MAP at 70 ± 5 mmHg. To assess the incidence of arterial rebound hypotension, NE infusion rates were again evaluated at 54 and 60 hours after randomization (i.e. 6 and 12 hours after termination of study drug infusion). None of the patients received further TP or AVP infusions.

### Statistical analysis

The primary endpoint of the present study was the reduction of average open-label NE requirements in patients treated with TP as compared with the AVP or NE group. To detect a 30% difference in NE infusion rates between groups, with an expected standard deviation (SD) of 25% and a test power of the analysis of variance (ANOVA) of 80%, a sample size of 15 individuals per group was required. Data are expressed as means ± SD, if not otherwise specified. Sigma Stat 3.10 software (SPSS, Chicago, IL, USA) was used for statistical analysis. After confirming normal distribution of all variables (Kolmogorov-Smirnov test), differences within and among groups were analyzed using a two-way ANOVA for repeated measurements with group and time as factors. Time-independent variables were compared with one-way ANOVA. In case of significant group differences over time, appropriate *post hoc *comparisons (Student-Newman-Keuls) were performed. Categorical data were compared using the chi-squared test. For all tests, an α-error probability of *P *< 0.05 was considered as statistically significant.

## Results

### Patients

Of the 119 screened septic shock patients who met the inclusion criteria of the study, 74 had to be excluded due to prior catecholamine therapy (n = 62), inappropriately low cardiac output (n = 7), chronic renal failure (n = 4), and severe liver dysfunction (n = 1). Finally, 45 consecutive patients were enrolled in the study and equally randomized to one of the three study groups (n = 15 per group; Figure 1). None of the enrolled patients died during the study period.

### Demographic data

Baseline characteristics including age, gender, body weight, origin of septic shock, and simplified acute physiology score II (SAPS II) are presented in Table 1. There were no significant differences in baseline characteristics between groups.

**Table 1:** Baseline characteristics, length of stay and outcome of the study patients

	TP (n = 15)	AVP (n = 15)	NE (n = 15)	*P *value
**Age, years**	67 (60; 71)	66 (60; 74)	64 (59; 72)	*0.889*
**Gender, male**	73%	67%	80%	*0.717*
**Body weight, kg**	85 (79; 100)	85 (71; 98)	85 (78; 90)	*0.612*
**SAPS II**	62 (57; 72)	60 (49; 66)	58 (52; 68)	*0.664*
**Cause of septic shock**	Necrotizing fasciitis (n = 1)	Endocarditis (n = 1)	Pancreatitis (n = 4)	*0.438*
	Pancreatitis (n = 3)	Necrotizing fasciitis (n = 2)	Peritonitis (n = 6)	
	Peritonitis (n = 5)	Peritonitis (n = 6)	Pneumonia (n = 5)	
	Pneumonia (n = 6)	Pneumonia (n = 6)		
**ICU mortality**	7/15	8/15	10/15	*0.533*
**ICU length of stay**	14 (9; 25)	17 (5; 27)	17(7; 23)	*0.878*

Data are given as median (25%; 75% range).

AVP = arginine vasopressin; ICU = intensive care unit; NE = norepinephrine; TP = terlipressin; SAPS II = simplified acute physiology score II.

### Norepinephrine and dobutamine requirements

**Figure 2:**
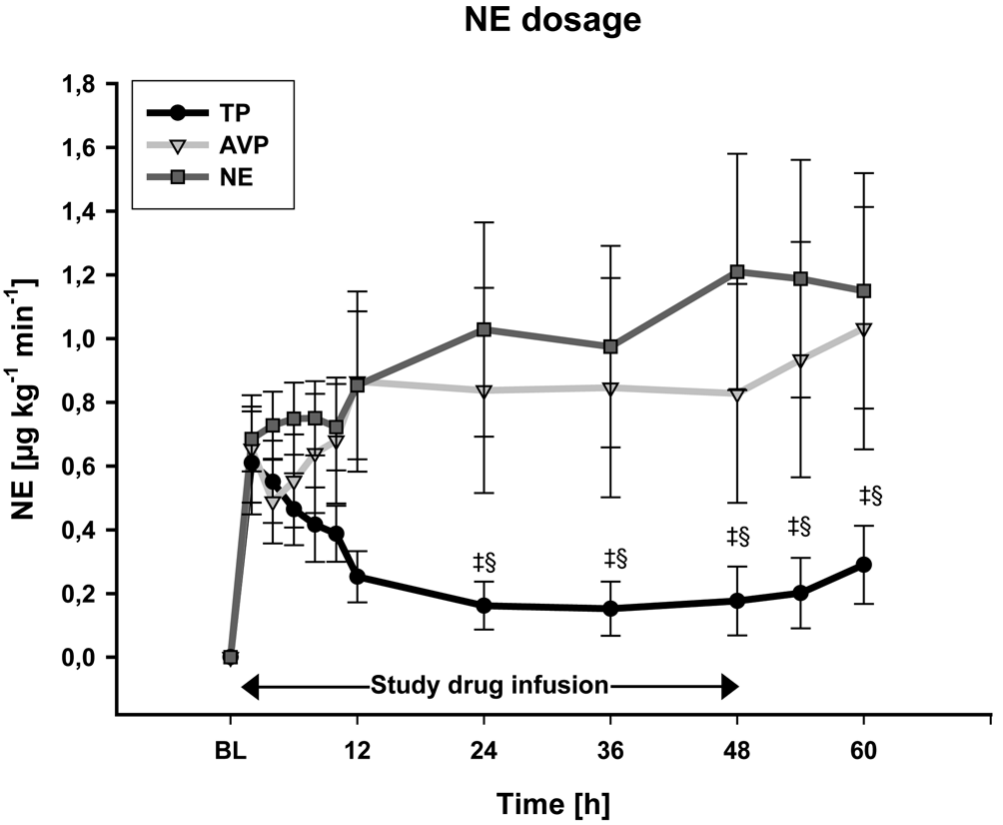
Norepinephrine requirements. AVP = arginine vasopressin; NE = norepinephrine; TP = terlipressin. ^‡ ^*P *< 0.05 vs. AVP (significant group effect); ^§ ^*P *< 0.05 vs. NE (significant group effect).

**Figure 3:**
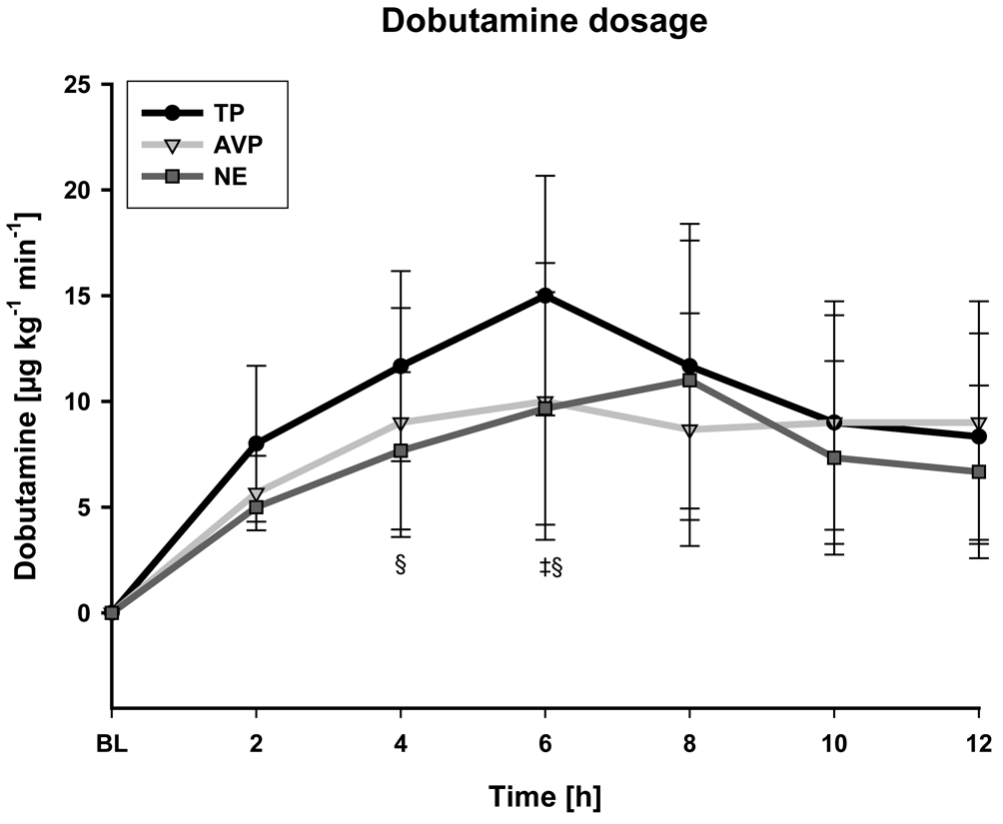
Dobutamine requirements. AVP = arginine vasopressin; MAP = mean arterial pressure; NE = norepinephrine; TP = terlipressin. ^‡^*P *< 0.05 vs. AVP (significant group effect); ^§ ^*P *< 0.05 vs. NE (significant group effect).

Open-label NE infusion rates increased over time in the AVP and NE groups (each *P *< 0.001 at 48 hours vs. baseline; Figure 2). Likewise, NE requirements increased during the first two hours of the study period in the TP group (*P *< 0.001). From 24 hours to the end of the intervention period, however, open-label NE infusion rates were significantly lower in the TP group as compared with the AVP and NE groups (*P *= 0.02 vs. AVP and *P *< 0.001 vs. NE at 48 hours). In addition, NE requirements were significantly higher 12 hours after discontinuation of the study drugs in the NE and AVP group as compared with the TP group (each *P *= 0.018 vs. AVP and NE at 60 hours). At six hours, dobutamine requirements were higher in TP-treated patients as compared with the other two groups. However, thereafter dobutamine doses were similar between groups during the first 12 hours of initial hemodynamic resuscitation (Figure 3). Activated protein C was administered in four patients in NE group and in five patients in both TP and AVP groups.

### Systemic hemodynamic variables

Systemic hemodynamic variables are summarized in Table 2. HR was significantly lower in the TP group as compared with the NE group over the whole interventional period (*P *= 0.047). There was no significant overall group difference in the other variables of systemic hemodynamics.

**Table 2:** Hemodynamic variables

	Baseline	12 hours	24 hours	36 hours	48 hours
**HR** (bpm)					
TP	95 ± 16	85 ± 19*	83 ± 21*	71 ± 14*^‡§^	71 ± 16*^‡§^
AVP	100 ± 22	98 ± 24	97 ± 27	92 ± 24^†^	93 ± 25^†^
NE	97 ± 21	92 ± 26	99 ± 29	95 ± 24^†^	96 ± 21^†^
**CI** (L/min/m)					
TP	4.0 ± 1.0	3.9 ± 1.0	3.7 ± 0.8	3.4 ± 0.6	3.5 ± 0.6
AVP	4.0 ± 1.1	4.2 ± 1.4	4.3 ± 1.1	3.9 ± 1.1	4.2 ± 1.9
NE	4.0 ± 1.0	3.9 ± 1.0	4.1 ± 1.1	3.9 ± 1.2	3.9 ± 1.5
**SVI** (mL/beats/m)					
TP	46 ± 13	46 ± 13	47 ± 12	48 ± 10	50 ± 10
AVP	41 ± 12	43 ± 12	46 ± 10	44 ± 12	47 ± 18
NE	43 ± 13	44 ± 14	44 ± 15	43 ± 16	42 ± 15
**MAP** (mmHg)					
TP	53 ± 6	70 ± 3*	71 ± 3*	72 ± 3*	71 ± 4*
AVP	53 ± 4	70 ± 3*	70 ± 3*	71 ± 3*	71 ± 3*
NE	54 ± 3	70 ± 4*	71 ± 2*	7 0 ± 3*	71 ± 3*
**MPAP** (mmHg)					
TP	25 ± 4	27 ± 4*	27 ± 4*	27 ± 5*	28 ± 5*
AVP	24 ± 4	28 ± 5*	28 ± 5*	28 ± 5*	29 ± 4*
NE	24 ± 7	28 ± 7*	29 ± 7*	29 ± 5*	30 ± 7*
**PAOP** (mmHg)					
TP	15 ± 2	17 ± 2	17 ± 2	17 ± 2	17 ± 2
AVP	15 ± 2	17 ± 2*	17 ± 4*	17 ± 3*	17 ± 2*
NE	15 ± 2	15 ± 2	16 ± 2	16 ± 2	16 ± 3
**RAP** (mmHg)					
TP	11 ± 3	12 ± 3	14 ± 3*	13 ± 3	13 ± 3
AVP	12 ± 3	15 ± 3*	14 ± 3*	15 ± 3*	15 ± 4*
NE	12 ± 3	13 ± 3	14 ± 3	14 ± 4	14 ± 4
**SVRI** (dyne·s/cm/m)					
TP	886 ± 291	1271 ± 334*	1287 ± 304*	1376 ± 241*	1348 ± 275*
AVP	861 ± 246	1157 ± 407*	1091 ± 325*	1235 ± 334*	1254 ± 531*
NE	874 ± 220	1237 ± 320*	1208 ± 348*	1266 ± 386*	1319 ± 471*
**PVRI** (dyne·s/cm/m)					
TP	196 ± 61	227 ± 99	219 ± 89	256 ± 108	260 ± 117
AVP	200 ± 83	243 ± 108	230 ± 128	254 ± 148	264 ± 134
NE	192 ± 115	266 ± 106*	275 ± 122*	298 ± 133*	313 ± 202*
**RVSWI** (g/m/beat)					
TP	8 ± 4	9 ± 4	8 ± 5	9 ± 4	10 ± 4
AVP	7 ± 3	8 ± 3	9 ± 3*	8 ± 3	9 ± 4*
NE	7 ± 3	9 ± 4*	9 ± 3*	8 ± 3*	9 ± 4*
**LVSWI** (g/m/beat)					
TP	22 ± 7	33 ± 9*	35 ± 10*	37 ± 8*	37 ± 9*
AVP	21 ± 7	31 ± 8*	32 ± 6*	33 ± 9*	34 ± 14*
NE	22 ± 7	33 ± 11*	33 ± 12*	32 ± 13*	31 ± 11*
**Fluids** (mL/24 h)					
TP			4833 ± 783		4353 ± 853
AVP			4860 ± 686		4513 ± 781
NE			4707 ± 860		4807 ± 853

AVP = arginine vasopressin; CI = cardiac index; HR = heart rate; LVSWI = left ventricular stroke work index; MAP = mean arterial pressure; MPAP = mean pulmonary arterial pressure; NE = norepinephrine; PAOP = pulmonary artery occlusion pressure; PVRI = pulmonary vascular resistance index; RAP = right atrial pressure; RVSWI = right ventricular stroke work index; SVI = stroke volume index; SVRI = systemic vascular resistance index; TP = terlipressin.

*P < 0.05 vs. baseline (significant time effect); ^† ^*P *< 0.05 vs. TP (significant group effect); ^‡^*P *< 0.05 vs. AVP (significant group effect); ^§^*P *< 0.05 vs. NE (significant group effect).

### New-onset tachyarrhythmias

The incidence of new-onset tachyarrhythmias (i.e atrial fibrillation) was 0 of 15 in the TP group, 1 of 15 in the AVP group and 4 of 15 in patients allocated to the control group (not significant; *P *= 0.054; chi-squared test).

### Acid-base homeostasis, oxygen transport variables

There were no significant overall differences between groups in any variable of acid-base homeostasis or oxygen transport, except for a lower pH and base excess as well as a higher arterial lactate concentration in the NE as compared with the TP group at 48 hours (Table 3).

**Table 3:** Oxygenation profile, acid-base variables and hemoglobin concentrations

	Baseline	12 hours	24 hours	36 hours	48 hours
**PH** (-log_10 _c(H^+^))					
TP	7.31 ± 0.1	7.32 ± 0.1	7.32 ± 0.1	7.34 ± 0.08	7.37 ± 0.08*
AVP	7.36 ± 0.09	7.35 ± 0.11	7.32 ± 0.12	7.34 ± 0.12	7.32 ± 0.11
NE	7.34 ± 0.1	7.34 ± 0.08	7.32 ± 0.08	7.31 ± 0.09	7.28 ± 0.12*^†^
**PaO_2_/FiO_2_**					
TP	176 ± 105	179 ± 82	189 ± 86	216 ± 95	220 ± 78
AVP	219 ± 118	231 ± 117	222 ± 129	211 ± 132	225 ± 133
NE	200 ± 97	216 ± 113	213 ± 101	194 ± 73	185 ± 85
**PaO_2_** (mmHg)					
TP	113 ± 44	127 ± 45	141 ± 47	164 ± 66	175 ± 64
AVP	123 ± 36	140 ± 48	130 ± 49	127 ± 59	139 ± 58
NE	120 ± 46	124 ± 44	125 ± 31	123 ± 34	114 ± 49
**pvO_2_** (mmHg)					
TP	36 ± 6	36 ± 6	36 ± 5	35 ± 5	36 ± 5
AVP	35 ± 6	38 ± 6	38 ± 5	38 ± 6	38 ± 6
NE	36 ± 7	38 ± 6	38 ± 6	39 ± 7	38 ± 6
**SaO_2_** (%)					
TP	96 ± 4	97 ± 3	98 ± 2	99 ± 2	99 ± 2
AVP	97 ± 3	98 ± 3	98 ± 2	96 ± 4	98 ± 2
NE	97 ± 2	96 ± 7	98 ± 1	98 ± 2	96 ± 7
**SvO_2_** (%)					
TP	60 ± 7	59 ± 11	60 ± 9	60 ± 8	63 ± 8
AVP	61 ± 12	65 ± 11	64 ± 7	63 ± 13	64 ± 12
NE	62 ± 10	66 ± 10	66 ± 9	66 ± 9	62 ± 12
**DO_2_I** (mL/min/m)					
TP	473 ± 105	468 ± 117	433 ± 92	393 ± 73	402 ± 70
AVP	464 ± 137	519 ± 189	550 ± 165	484 ± 123	520 ± 242
NE	460 ± 131	471 ± 157	482 ± 136	462 ± 136	467 ± 162
**VO_2_I** (mL/min/m)					
TP	184 ± 58	184 ± 49	171 ± 32	160 ± 43	152 ± 38
AVP	173 ± 51	173 ± 59	193 ± 65	168 ± 52	173 ± 51
NE	163 ± 41	147 ± 35	160 ± 57	153 ± 51	164 ± 67
**O_2_-ER** (%)					
TP	38 ± 6	40 ± 9	40 ± 8	41 ± 8	38 ± 8
AVP	39 ± 12	35 ± 9	35 ± 6	36 ± 11	37 ± 10
NE	37 ± 10	32 ± 6	34 ± 9	34 ± 9	36 ± 10
**PaCO_2_** (mmHg)					
TP	45 ± 6	42 ± 6	41 ± 9	40 ± 8	38 ± 6
AVP	43 ± 9	40 ± 5	42 ± 4	41 ± 6	41 ± 6
NE	44 ± 9	43 ± 9	44 ± 8	44 ± 8	43 ± 9
**ABE** (mmol/L)					
TP	-2.9 ± 5.1	-4.6 ± 4.2	-4.9 ± 4.6	-4.0 ± 4.2	-3.1 ± 4.2
AVP	-1.8 ± 6.5	-2.8 ± 7.0	-3.7 ± 6.7	-1.6 ± 7.8	-4.1 ± 6.5
NE	-2.5 ± 4.5	-2.5 ± 4.3	-3.5 ± 4.3	-4.2 ± 3.9	-6.2 ± 5.4*
**Arterial lactate** (mmol/L)					
TP	3.1 ± 1.8	2.9 ± 1.9	2.9 ± 2.0	3.4 ± 2.4	3.6 ± 3.0
AVP	3.0 ± 2.4	3.2 ± 2.3	3.4 ± 2.3	3.2 ± 2.3	3.4 ± 3.3
NE	3.1 ± 2.2	3.3 ± 2.8	3.4 ± 2.8	3.6 ± 2.4	4.3 ± 3.4*
**Hemoglobin** (g/dL)					
TP	8.6 ± 0.9	8.7 ± 0.7	8.4 ± 1.2	8.2 ± 0.6^‡^	8.1 ± 0.6
AVP	8.3 ± 0.9	8.7 ± 1.1	9 ± 1.1*	9 ± 1*^†^	8.8 ± 0.9
NE	8.3 ± 0.8	8.7 ± 0.9	8.4 ± 0.5	8.5 ± 0.8	8.9 ± 1

ABE = arterial base excess; AVP = arginine vasopressin; DO_2_I = oxygen delivery index; NE = norepinephrine; O_2_-ER = oxygen extraction rate; PaCO_2 _= partial pressure of arterial carbon dioxide; PaO_2_/FiO_2 _= ratio of oxygen tension over inspired oxygen concentration; PaO_2 _= partial pressure of arterial oxygen; pH = arterial pH; pvO_2 _= mixed venous oxygen tension; SaO_2 _= arterial oxygen saturation; SvO_2 _= mixed venous oxygen saturation; VO_2_I = oxygen consumption index; TP = terlipressin.

* *P *< 0.05 vs. baseline (significant time effect); ^† ^*P *< 0.05 vs. TP (significant group effect); ^‡ ^*P *< 0.05 vs. AVP (significant group effect); ^§ ^*P *< 0.05 vs. NE (significant group effect).

### Regional hemodynamics

There were no significant overall differences between groups in any variable of regional hemodynamics. Nevertheless, a time-dependent decrease in PDR and CBI was observed in the AVP and NE groups (both *P *< 0.05 at 48 hours vs. baseline; Table 4).

**Table 4:** Regional hemodynamics

	Baseline	12 hours	24 hours	36 hours	48 hours
**CBI** (mL/min/m)					
TP	353 ± 226	387 ± 202	367 ± 179	299 ± 139	351 ± 129
AVP	397 ± 171	409 ± 209	367 ± 222	308 ± 185*	305 ± 201*
NE	327 ± 135	358 ± 173	312 ± 145	271 ± 136	252 ± 206
**PDR** (%)					
TP	13 ± 6	14 ± 5	13 ± 4	12 ± 5	13 ± 4
AVP	15 ± 5	15 ± 6	13 ± 7	12 ± 7*	11 ± 6*
NE	14 ± 5	13 ± 5	12 ± 6	11 ± 6*	10 ± 7*
**P_g-a_CO_2_** (mmHg)					
TP	23 ± 11	24 ± 8	22 ± 6	20 ± 6	20 ± 7
AVP	25 ± 7	28 ± 8	27 ± 9	28 ± 12	28 ± 10
NE	24 ± 11	28 ± 10	28 ± 8	26 ± 8	31 ± 12
**Urinary output** (mL/h)					
TP	34.6 ± 31.3	69.3 ± 70.4	49.2 ± 49.5	48.5 ± 41.4	46.6 ± 33.3
AVP	42.3 ± 46.9	42 ± 39	42 ± 41.6	40.7 ± 45.7	43.3 ± 58.7
NE	38.6 ± 34.3	55.4 ± 74.1	66 ± 77	58.6 ± 56.1	58.6 ± 63.8

AVP = arginine vasopressin; CBI = blood clearance of indocyanine green; NE = norepinephrine; PDR = plasma disappearance rate of indocyanine green; P_g-a_CO_2 _= gastric-mucosal arterial carbon dioxide partial pressure difference; TP = terlipressin.

* *P *< 0.05 vs. baseline (significant time effect); ^† ^*P *< 0.05 vs. TP (significant group effect); ^‡ ^*P *< 0.05 vs. AVP (significant group effect); ^§ ^*P *< 0.05 vs. NE (significant group effect).

### Variables of organ function and injury

Variables of organ function and coagulation were similar between groups (Table 5), except for BILT and BILD, which were significantly higher in the AVP and NE group as compared with patients treated with TP at the end of the 48-hour intervention period (BILT: TP vs. NE, *P *= 0.001; TP vs. AVP, *P *= 0.009; BILD: TP vs. NE, *P *= 0.002; TP vs. AVP, *P *= 0.013).

**Table 5:** Surrogate variables of organ function and injury

	Baseline	12 hours	24 hours	36 hours	48 hours
**Creatinine** (mg/dL)					
TP	2.5 ± 1	2.6 ± 1.2	2.8 ± 1.4	2.8 ± 1.3	2.8 ± 1.4
AVP	2.2 ± 1	2.4 ± 1.1	2.4 ± 1.2	2.5 ± 1.4	2.4 ± 1.2
NE	2.2 ± 1.6	2.6 ± 1.7	2.7 ± 1.7*	2.9 ± 1.8*	3.3 ± 2*
**Creatinine, rel**. (%)					
TP	--	4 ± 16	11 ± 23	13 ± 27	14 ± 35^§^
AVP	--	11 ± 17	12 ± 26	15 ± 31	10 ± 21^§^
NE	--	19 ± 23	23 ± 37	34 ± 48	54 ± 77^†‡^
**Bilirubin, tot**. (mg/dL)					
TP	1.2 ± 0.7	1 ± 0.5	1 ± 0.5	0.9 ± 0.3^‡§^	0.9 ± 0.3^‡§^
AVP	1.6 ± 1.3	1.6 ± 1.2	1.8 ± 1.6	2.0 ± 2.0^†^	2.3 ± 2.8^†^
NE	1.6 ± 0.9	1.9 ± 0.8	2.0 ± 1.2	2.3 ± 1.7*^†^	2.8 ± 2.5*^†^
**Bilirubin, dir**. (mg/dL)					
TP	0.5 ± 0.3	0.5 ± 0.4	0.5 ± 0.3	0.4 ± 0.2^§^	0.3 ± 0.1^‡§^
AVP	0.8 ± 0.9	1 ± 1.2	1.1 ± 1.4	1.1 ± 1.5	1.4 ± 1.9*^†^
NE	0.8 ± 0.5	1.2 ± 0.8	1.2 ± 0.9	1.6 ± 1.7*^†^	1.9 ± 2.1*^†^
**ASAT** (U/L)					
TP	52 ± 26	52 ± 33	54 ± 35	53 ± 42	48 ± 34
AVP	63 ± 49	85 ± 57	79 ± 66	119 ± 163	91 ± 95
NE	72 ± 68	89 ± 115	95 ± 132	103 ± 146	90 ± 122
**ALAT** (U/L)					
TP	30 ± 14	34 ± 15	33 ± 14	35 ± 18	30 ± 13
AVP	45 ± 31	58 ± 42	63 ± 53	69 ± 67	81 ± 85
NE	43 ± 45	62 ± 80	68 ± 103	73 ± 114	63 ± 97
**Amylase** (U/L)					
TP	168 ± 97	148 ± 90	133 ± 79	127 ± 79	144 ± 124
AVP	165 ± 111	152 ± 100	143 ± 79	147 ± 110	123 ± 70
NE	203 ± 191	199 ± 182	217 ± 221	206 ± 212	172 ± 152
**Lipase** (U/L)					
TP	138 ± 145	125 ± 92	97 ± 64	144 ± 126	116 ± 91
AVP	133 ± 90	124 ± 68	127 ± 64	136 ± 72	134 ± 65
NE	134 ± 160	198 ± 282	120 ± 96	158 ± 162	115 ± 65
**Troponine I** (ng/mL^)^					
TP	0.31 ± 0.3	0.31 ± 0.51	0.22 ± 0.41	0.19 ± 0.35	0.18 ± 0.3
AVP	0.56 ± 1	0.84 ± 1.5	1.23 ± 2.4	1.37 ± 2.5	1.17 ± 2
NE	0.58 ± 0.9	0.66 ± 0.8	0.72 ± 0.75	0.69 ± 0.86	0.63 ± 1
**Platelet count** (10^3 ^cells/μL)					
TP	119 ± 68	103 ± 59	93 ± 59*	78 ± 48*	73 ± 41*
AVP	110 ± 56	102 ± 63	95 ± 53	95 ± 55	93 ± 50
NE	114 ± 64	102 ± 52	99 ± 58	94 ± 60	94 ± 69
**INR**					
TP	1.4 ± 0.2	1.4 ± 0.2	1.4 ± 0.2	1.4 ± 0.2	1.4 ± 0.2
AVP	1.5 ± 0.5	1.5 ± 0.3	1.5 ± 0.5	1.5 ± 0.4	1.5 ± 0.5
NE	1.5 ± 0.3	1.5 ± 0.3	1.4 ± 0.4	1.3 ± 0.2	1.4 ± 0.3
**APTTr**					
TP	1.7 ± 0.6	1.6 ± 0.5	1.6 ± 0.4	1.8 ± 0.6	1.7 ± 0.7
AVP	1.5 ± 0.5	1.5 ± 0.6	1.6 ± 0.7	1.6 ± 0.7	1.7 ± 0.7
NE	1.5 ± 0.2	1.4 ± 0.2	1.4 ± 0.2	1.4 ± 0.2	1.6 ± 0.3

ALAT = alanine aminotransferase; aPTTr = activated partial thromboplastin time ratio; ASAT = aspartate aminotransferase; AVP = arginine vasopressin; Creatinine rel = relative increase in creatinine concentrations from baseline; INR = international normalized ratio; NE = norepinephrine; TP = terlipressin.

* *P *< 0.05 vs. baseline (significant time effect); ^† ^*P *< 0.05 vs. TP (significant group effect); ^‡ ^*P *< 0.05 vs. AVP (significant group effect); ^§ ^*P *< 0.05 vs. NE (significant group effect).

Creatinine plasma concentrations increased with time only in the NE group (*P *< 0.001 at 48 hours vs. baseline). The relative increase in creatinine concentrations over the 48-hour intervention period was significantly higher in the NE group as compared with the TP and AVP group (each *P *< 0.001). Whereas 4 of 15 (26.7%) and 5 of 15 (33.3%) patients required renal replacement therapy at the end of the study period in the TP and AVP group, respectively, 8 of 15 patients (53.3%) required renal replacement therapy at the end of the study period in the NE group (n.s.; *P *= 0.293; chi-squared test). There were no differences in coagulation variables except for a time-dependent decrease in platelet count in the TP group (*P *< 0.001 at 48 hours vs. baseline).

### Markers of systemic inflammation

IL-6 concentrations significantly decreased in the AVP group (*P *= 0.044 at 48 hours vs. baseline), and there was a strong tendency towards a decrease in the TP group (*P *= 0.052 at 48 hours vs. baseline). However, there were no significant differences in TNF-α or IL-1β concentrations among groups (Table 6).

**Table 6:** Markers of systemic inflammation

	Baseline	48 hours
**IL-6** (pg/mL)		
TP	612 ± 640	296 ± 367
AVP	621 ± 595	293 ± 324 *
NE	655 ± 585	380 ± 251
**IL-1β** (pg/mL)		
TP	6.6 ± 0.6	6.1 ± 0.6
AVP	6.7 ± 1	6.5 ± 1
NE	6.5 ± 0.7	6.6 ± 0.7
**TNF-α** (pg/mL)		
TP	24 ± 21	18 ± 6
AVP	24 ± 16	24 ± 27
NE	28 ± 15	29 ± 21
**Temperature** (°C)		
TP	38.6 ± 2	37.8 ± 0.8
AVP	39 ± 0.4	38.2 ± 1
NE	38.8 ± 0.2	38.5 ± 0.8

AVP = arginine vasopressin; NE = norepinephrine; TP = terlipressin.

* *P *< 0.05 vs. baseline (significant time effect).

### Length of ICU stay and outcome

Length of ICU stay and ICU mortality were similar between groups (Table 1).

## Discussion

The major findings of the present study are that continuous, low-dose TP infusion at the investigated dose was effective in reversing sepsis-induced arterial hypotension and in reducing NE requirements.

In the current clinical trial, TP, AVP and NE – when administered as first-line vasopressor agents – were effective in increasing MAP to goal values of 70 ± 5 mmHg when combined with open-label NE. The vasoconstrictive effects of AVP and TP mainly depend on V_1 _receptor stimulation. Nevertheless, AVP may also exert vasodilatory effects in a dose-dependent manner, possibly mediated by nitric oxide liberation secondary to stimulation of V_2 _receptors [22]. In this context, Barrett and colleagues [23] recently reported that the selective V_1 _agonist F-180 is a more effective vasoconstrictor agent as compared with AVP. The latter observation is in accordance with the finding of the present study that TP, a relatively selective V_1 _agonist as compared with AVP (V_1_:V_2 _ratio of 2.2:1 vs. 1:1) [22], enabled a marked reduction in open-label NE requirements. As expected, due to its effective half-life of four to six hours, we noticed a longer duration of the TP effects (i.e. lack of rebound hypotension) [22].

The somewhat surprising observation of the present study that AVP only tended to but did not significantly reduce NE requirements is in contrast with the results of VASST (which used an identical vasopressin dose), in which AVP administration allowed a reduction in NE requirements [5]. However, there are several reasons that might explain this discrepancy. First, the considerably higher sample size of VASST as compared with the present study makes it more likely to detect significant differences. Moreover, in VASST [5], MAP at baseline was 72 to 73 mmHg, whereas it was considerably lower in the present study. Second, the mean time elapsed from meeting the criteria for study entry to infusion of AVP was 12 hours in VASST [5]. By contrast, in our study, a different hemodynamic condition at baseline (i.e. arterial hypotension), as well as the administration of AVP as a first-line therapy could have played a pivotal role in this regard [4]. In addition, the lack of reduction in NE requirements may potentially be explained by the low dose infused in the present study (0.03 U/min). Although previous studies suggest that AVP infusion in septic shock should not exceed 0.04 U/min because of the potential risk of adverse effects [3,24], Luckner and colleagues [25] recently reported that 0.067 U/min is more effective in hemodynamic support and catecholamine reduction than 0.033 U/min. Finally, it has to be underlined that this specific dose has not yet been investigated as first-line therapy in the treatment of human septic shock. Therefore, it is possible that in the present study, TP was more effective than AVP because the TP dose was relatively higher as compared with the vasopressin dose.

In harmony with previous experimental and clinical studies [11-14], we did not notice a decrease in CI, DO_2_I and SvO_2 _following low-dose AVP or TP infusion in fluid resuscitated septic shock patients. In this regard, it is important to underline that dobutamine doses administered to achieve SvO_2 _values of 65% or moreduring the initial phase of hemodynamic resuscitation were similar between groups. In addition, neither AVP nor TP negatively affected pulmonary hemodynamics and function, as suggested by constant PVRI values and partial pressure of arterial oxygen (PaO_2_)/fraction of inspired oxygen (FiO_2_) ratio. These findings confirm the theory that continuous TP infusion may be favourable over TP bolus infusion, because the latter approach has been reported to excessively increase SVRI and PVRI, as well as to decrease HR and CI [11].

Previous studies investigating low-dose AVP or TP in patients with septic shock following adequate fluid resuscitation reported few or no unwanted side effects within the splanchnic circulation [7,26-29]. In agreement with these previous studies, we did not find significant overall differences among groups in terms of arterial lactate concentrations or acid-base homeostasis, as well as surrogate markers of splanchnic perfusion. The absence of detrimental hepatosplanchnic hemodynamic effects of TP and AVP during the observation period is further confirmed by the lack of significant overall differences among groups in terms of liver and pancreatic enzymes. Nevertheless, at the end of the study period, both BILT and BILD were significantly higher in both the AVP and NE group as compared with patients treated with TP. The increase in BILT in the AVP group noticed in the present study is in agreement with previous studies [25,27,30] reporting similar findings after AVP administration. In contrast, we did not find any differences in BILT 48 hours after TP administration. It has been postulated that AVP might contribute to an increase in BILT concentrations by a reduction of biliary output and bile flow after an initial transient increase [31]. In addition, it has been shown that AVP may modulate hepatocyte tight junctional permeability and thus produce cholestasis [32]. Although speculative, it is possible that these effects are less pronounced when TP is administered, probably due to its higher V_1 _selectivity. Nevertheless, the implication of this finding for the course of the disease remains uncertain and should be clarified in future studies.

Although AVP may contribute to antidiuresis in a dose-dependent manner [33], recent studies revealed that in the presence of septic shock, vasopressin analogues may increase diuresis and improve renal function [7-9,24,26,28,29]. Different pharmacological effects on the afferent and efferent arterioles [34], as well as the pathophysiological features in vasopressin receptor physiology in sepsis [35] may account for these observations [7-9,24,26,28,29]. Moreover, the AVP-associated increase in systemic blood pressure may contribute to an increase in urine output [36]. Notably, a *post hoc *analysis of the VASST data [37] demonstrated a reduced rate of progression to acute renal failure in patients at risk for acute renal failure ('R', according to the RIFLE criteria [38]) treated with AVP. In harmony with the latter observation [37], neither AVP nor TP negatively affected renal function in the present study.

AVP has been reported to activate platelets via V_1 _receptors, leading to an increase in CD62 expression [39,40] and a decrease in platelet count in patients with normal platelets, but not in patients with low platelets [39]. In this context, it is another interesting finding of the present study that TP, as compared with AVP and NE, significantly decreased platelet count. However, in accordance with a previous study [40], neither AVP nor NE negatively affected the coagulation system.

The present study has some limitations that we would like to acknowledge. First, because there are no equivalent doses or data comparing different doses of AVP and TP, we decided to evaluate the efficacy of fixed doses of the study drugs in reaching the threshold MAP and to investigate their effects on open-label NE requirements. We therefore chose the AVP dose investigated in VASST (i.e. 0.03 U/min of AVP and 15 μg/min of NE) [5] and a low TP dose previously reported to be safe and effective in a case series [13]. In this regard, it needs to be considered that AVP was administered at a fixed dose of 0.03·U/min. It might be argued that a weight-adjusted TP dose was compared with a fixed AVP dose and thus the chosen doses might not have been pharmacologically equivalent. Therefore, it is possible that the TP dose was relatively higher as compared with the AVP dose.

Second, we performed a pilot study with the reduction of open-label NE requirements as the primary endpoint. In this regard, it has to be underlined that there is no reliable evidence that a reduction in catecholamine requirements may lead to an improved outcome. Third, we investigated only a small number of septic shock patients treated over a relative brief period. In this regard, the risk of positive results in a study with numerous secondary variables and time points has to be taken into account. Thus, caution should be exercised in interpreting the results of the secondary outcome variables. Properly powered, randomized controlled trials are required to determine the effects of TP infusion on clinical outcome. All patients included in the present study received hydrocortisone, so we cannot judge if and how corticosteroids affected our results [41,42]. For safety reasons, we opted for a 48-hour intervention period, because it was impossible to measure the circulating levels of TP. Although there is no evidence of drug accumulation over time, we cannot rule out this possibility when TP is infused over a more prolonged period. Moreover, hepatosplanchnic perfusion was assessed using PDR and CBI. Although PDR and CBI have been found to be a good predictor of survival in critically ill patients, at best it reflects the total splanchnic blood flow without separating hepatic arterial from portal venous flow. In addition, mucosal blood flow was estimated by gastric tonometry, a methodology that does not necessarily reflect changes in other parts of the gastrointestinal tract.

## Conclusions

Taken together, our results demonstrate that a continuous infusion of a relatively low dose of TP (1.3 μg/kg/h) was effective in reversing sepsis-induced hypotension and in reducing NE requirements. Larger randomized controlled clinical trials are necessary to explicitly clarify whether or not low-dose TP infusion may improve the overall outcome of patients with septic shock as compared with standard therapy. Awaiting these results, continuous TP infusion should not be routinely used outside the scope of controlled clinical trials and might be considered as a rescue therapy, when catecholamines are no longer effective.

## Key messages

• Continuous infusion of low-dose TP – when given as first-line vasopressor agent in septic shock – reduces open-label NE requirements.

• Low-dose AVP or TP infusion do not decrease in CI, DO_2_I and SvO_2 _in adequately fluid resuscitated septic shock patients.

• Continuous TP infusion may be favourable over TP bolus infusion, because the latter approach has been reported to excessively increase SVRI and PVRI as well as decreases in HR and CI.

• Neither AVP nor TP negatively affected pulmonary hemodynamics and function.

• There are no differences between TP, AVP and NE in terms of regional hemodynamics or acid-base homeostasis when they are administered as first-line vasopressor agent in septic shock.

## Abbreviations

ANOVA: analysis of variance; AVP: arginine vasopressin; BILD: direct bilirubin; BILT: total bilirubin; CBI: blood clearance of indocyanine green related to body surface area; CI: cardiac index; DO_2_I: systemic oxygen delivery index; FiO_2_: fraction of inspired oxygen; HR: heart rate; ICU: intensive care unit; IL: interleukin; LVSWI: left ventricular stroke work index; MAP: mean arterial pressure; MPAP: mean pulmonary arterial pressure; NE: norepinephrine; O_2_-ER: oxygen extraction rate; PaO_2_: partial pressure of arterial oxygen; PAOP: pulmonary arterial occlusion pressure; PDR: plasma disappearance rate of indocyanine green; PVRI: pulmonary vascular resistance index; RAP: right atrial pressure; RVSWI: right ventricular stroke work index; SAPS II: Simplified Acute Physiology Score II; SD: standard deviation; SvO_2_: mixed-venous oxygen saturation; SVRI: systemic vascular resistance index; TNF: tumor necrosis factor; TP: terlipressin; VASST: Vasopressin and Septic Shock Trial; VO_2_I: systemic oxygen consumption index.

## Competing interests

The authors declare that they have no competing interests.

## Authors' contributions

AM and MW were responsible for the study design and coordination and drafted the manuscript. CE, ML, SR and HVA participated in the design of the study, performed the statistical analysis and helped to draft the manuscript. AO and VC participated in the study design and helped to draft the manuscript. AB and MD participated in the study design, performed laboratory measurements and helped to draft the manuscript. PP participated in the study design and coordination and helped to draft the manuscript and obtained funding. All authors listed on the title page read and approved the final manuscript.
